# Structure, Distribution, Chemical Composition, and Gene Expression Pattern of Glandular Trichomes on the Leaves of *Rhus potaninii* Maxim

**DOI:** 10.3390/ijms22147312

**Published:** 2021-07-07

**Authors:** Qin Lu, Nawaz Haider Bashir, Hai-Xia Wu, Weiwei Wang, Jinwen Zhang, Yongzhong Cui, Hang Chen

**Affiliations:** 1 Research Institute of Resource Insects, Chinese Academy of Forestry, Kunming 650224, China; lqin@caf.ac.cn (Q.L.); nawazhaider@caf.ac.cn (N.H.B.); Haixiawu@caf.ac.cn (H.-X.W.); sk121www@caf.ac.cn (W.W.); jinwen@caf.ac.cn (J.Z.); cafkmcyz@126.com (Y.C.); 2 The Key Laboratory of Cultivating and Utilization of Resources Insects of State Forestry Administration, Kunming 650224, China

**Keywords:** distribution pattern, gene expression pattern, glandular trichome, histological structure, secretion, plant defense

## Abstract

*Rhus potaninii* Maxim is an economically and medicinally important tree species in China. It produces galls (induced by aphids) with a high abundance of tannins. Here, we discuss the histology, cellular structures and their distribution, and the macromolecular components of secretive glandular trichomes on the leaves of *R. potaninii*. A variation in the density of glandular trichomes and tomenta was found between the adaxial and abaxial sides of a leaf in different regions and stages of the leaf. The glandular trichomes on *R. potaninii* trees comprise a stalk with no cellular structure and a head with 8–15 cells. Based on staining, we found that the secretion of glandular trichomes has many polysaccharides, phenolic compounds, and acidic lipids but very few neutral lipids. The dense glandular trichomes provide mechanical protection for young tissues; additionally, their secretion protects the young tissues from pathogens by a special chemical component. According to transcriptome analysis, we found enhanced biosynthetic and metabolism pathways of glycan, lipids, toxic amino acids, and phenylpropanoids. This shows a similar tendency to the staining. The numbers of differentially expressed genes were large or small; the averaged range of upregulated genes was greater than that of the downregulated genes in most subpathways. Some selectively expressed genes were found in glandular trichomes, responsible for the chitinase activity and pathogenesis-related proteins, which all have antibacterial activity and serve for plant defense. To our knowledge, this is the first study showing the components of the secretion from glandular trichomes on the leaf surface of *R. potaninii*.

## 1. Introduction

Plants possess several microstructures which are not visible to the eyes. These structures usually play important roles in different stages of plant life. For instance, stomata are responsible for gas exchange in plants, i.e., in respiration and photosynthesis [1]. Another important accessory structure of plant is trichomes. Plant trichomes can be classified as glandular trichomes (GTs) and nonglandular trichomes. Nonglandular trichomes are also called tomenta, which are responsible for providing mechanical protection for plants against pests such as aphids; they also guide pollinators to collect pollen and affect photosynthesis, leaf temperature, and water loss through increased light reflectance in desert species [2,3]. GTs are common on the leaf surface, flowers, and stems in approximately 30% of all vascular plants [4]. GTs exist in different forms, such as peltate, capitate, and ball forms [5]. They exhibit tremendous species-specific characteristics [6]. The cellular structures of GTs are different in various species. Some GTs consist of a globular base with multiple cells and a small head with a single glandular cell; some consist of 4–8 cells and a noncellular stalk, while some have just a few cells without a stalk [7,8]. 

Many plants have their distinctive looks and smells due to the secretion from GTs; for example, the specific smells of *Solanum lycopersicum* L. and *Mentha haplocalyx* Briq are caused by geranylacetone, piperitol, piperitone, and more than 30 other compounds [9,10]. These smells prevent or repel arthropods or even some large herbivores from eating them [11]. In addition to the chemical protection, the secretions of GTs work as a protective layer of young leaves or other developing organs which serve as barriers against extreme weather (e.g., drought) and other stresses (e.g., attack from pathogens and pests) [4]. Besides their importance to plants, secondary metabolites secreted by GTs have many uses in our daily life. For instance, artemisinin, a sesquiterpene lactone produced in GTs of annual wormwood (*Artemisia annua* Linn.), is used for the treatment of malaria [12]. The uniqueness of GTs in different plant species give them important characteristics concerning the identity and classification of plant species [13].

*Rhus potaninii* Maxim. is a tree species with great economic value in China. The leaves of *R. potaninii* trees can produce galls after induction by aphids (*Kaburagia rhusicola*) feeding during the initial stage [14]. The gall tissues contain a very high content of various tannins, which have been used in medical and chemical industries for centuries in China [15]. At present, the cultivation of *R. potaninii* trees and harvesting of tannin-rich galls remain a vital source of income in some villages in southern China. Most importantly, the content and transcriptome of GTs in *R. potaninii* have not been previously recorded. The objective of this study is to examine the histology, cellular structures and their distribution, and the macromolecular composition of trichomes on the leaves of *R. potaninii* trees at different stages and the transcriptome of mature GTs. Our observations provide useful information for further studying the interactions between *R. potaninii* and its surroundings.

## 2. Results

### 2.1. Morphological Characteristics and the Distribution of GTs

**Figure 1:**
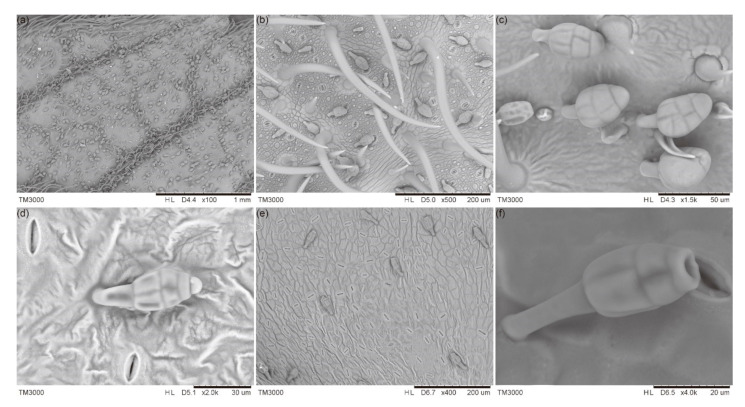
Appearance of GTs under an SEM. (**a**) Dense GTs on the adaxial side of a leaf. (**b**) Enlarged young and mature GTs on leaves during early stages (on the abaxial side of a leaf). (**c**) Plump-like structures of mature GTs on leaves during the second stage. (**d**) A GT that was secreting. (**e**) Physical appearance of thinner GTs on leaves during the fourth stage. (**f**) A view of the top of a GT, which became hollow after secreting.

**Figure A1:**
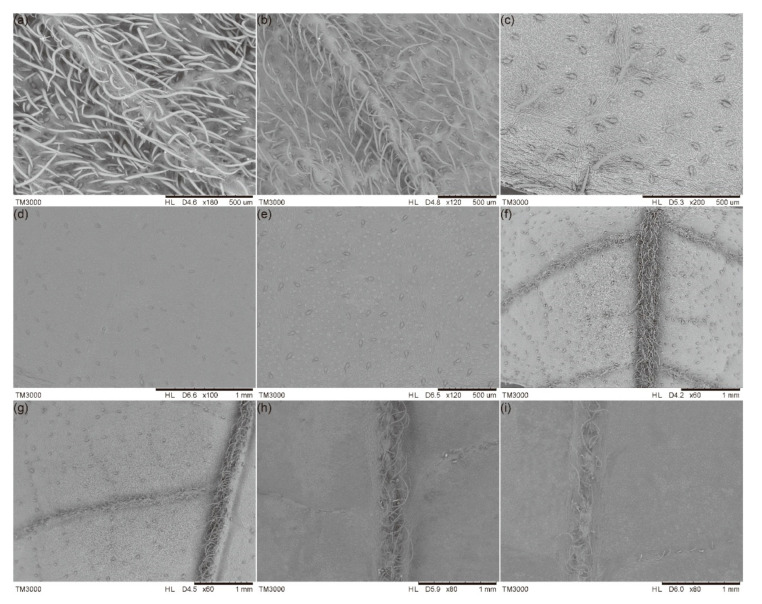
SEM scanning pictures of trichomes on leaves at different stages. (**a**–**e**) Images of abaxial site of leaves at the first to the fifth stage, respectively. (**f**–**i**) Images of the adaxial side of leaves during the second to the fifth stage of leaf development, respectively.

Dense GTs were found on both the adaxial and abaxial sides of young leaves on *R. potaninii* trees (Figure 1a,b). Mature GTs were plump structures consisting of a stalk and a head during the initial stages (first to third). The heads were divided into several parts through grooves, which appeared as interspaces between cells under a scanning electron microscope (SEM) (Figure 1c). Besides these grooves, the surface of GTs was fairly smooth (Figure 1b,c). During a later stage (third stage), the secretion was present on the top of the head (Figure 1d and Figure A1), and the GT’s head became longer and thinner (Figure 1e). After secreting, the top of the GTs became hollow (Figure 1e,f). No new GTs were found from the second stage.

There was no accessory structure around the base of a GT, but we found a petaloid structure around the base of a young tomentum (Figure 1c). 

**Figure 2:**
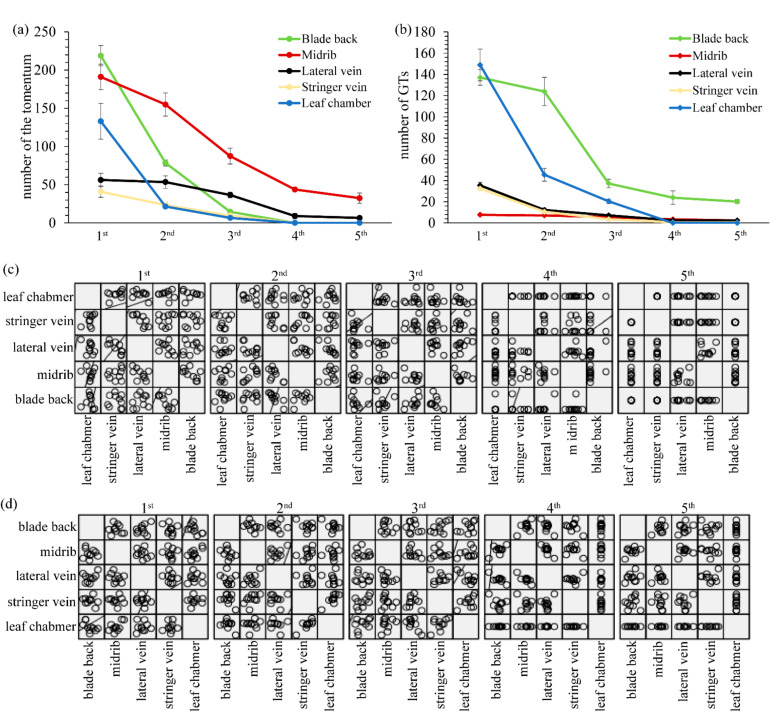
The number and multivariate analysis of variance (MANOVA) of tomenta and GTs during different stages. (**a**) The number of tomenta during different stages. (**b**) The number of GTs at different stage. (**c**) The MANOVA of tomenta. (**d**) The MANOVA of GTs. *p* < 0.01.

The distribution of GTs and tomenta was consistent on both the adaxial and abaxial sides of a leaf. The number of GTs and tomenta decreased during leaf development. On the back of the leaf, GTs and tomenta were randomly distributed around the vein and leaf chamber, with tomenta around 214.69 ± 15.76/mm^2^ (1st), 81.05 ± 10.29/mm^2^ (2nd), 14.31 ± 2.00/mm^2^ (3rd), 0.07 ± 0.01/mm^2^ (4th), and 0/mm^2^ (5th) at different stages (Figure 2a). The number of GTs ranged between 139.64 ± 7.37/mm^2^ (1st), 124.29 ± 15.53/mm^2^ (2nd), 39.59 ± 4.10/mm^2^ (3rd), 20.8 ± 10.40/mm^2^ (4th), and 19.99 ± 1.82/mm^2^ (5th) at different stages (Figure 2b and Figure A1a–e). The distribution of GTs and tomenta was different on the adaxial side of the leaf. The density of tomenta around the midrib was 192.96 ± 18.10/mm, 145.70 ± 13.84/mm, 93.31 ± 15.03/mm, 44.35 ± 0.21/mm, and 31.86 ± 9.39/mm at different stages, respectively (Figure 2a). On the other hand, the density of GTs was 7.77 ± 0.81/mm, 7.52 ± 0.59/mm, 5.12 ± 0.21/mm, 3.43 ± 1.07/mm, and 2.16 ± 0.76/mm at different stages, respectively (Figure 2b). A similar distribution was observed, but the ratio between GTs and tomenta increased around the leaf chamber and stringer vein in the second and third leaf stages. No GTs and tomenta were found in the later stages of leaf chambers (Figure 2a,b and Figure A1h,i). In general, the density of tomenta in young leaves was midrib > leaf chamber > lateral vein > stringer vein. At later stages, the density of tomenta was midrib > lateral vein > stringer vein > leaf chamber. No tomentum was found on the backside of the leaves during the fifth stage (Figure 2a and Figure A1e). However, the density of GTs showed a completely different distribution pattern on the adaxial side. On young leaves (first and second stages), the density of GTs was leaf chamber > lateral vein > stringer vein > midrib. The pattern changed to leaf chamber > lateral vein > midrib > stringer vein in third stage leaves; to midrib > lateral vein > stringer vein in fourth stage leaves; and to vein > midrib > stringer vein > leaf chamber in fifth stage leaves (Figure 1a, Figure 2b and Figure A1f–i).

Multivariate analysis of variance (MANOVA) showed that tomenta and GTs in different regions of different stages had no linear relation except the number of tomenta between the abaxial side and the stringer vein from the first to fourth stages. The number of tomenta and GTs in different regions showed significant differences and were significantly positively correlated with location (*p* < 0.01) (Figure 2c,d). The GTs were more likely to be distributed on the back of the blade and leaf chamber, but the midrib possessed more tomenta throughout the growth stage.

### 2.2. The Histological Structure of GTs

**Figure 3:**
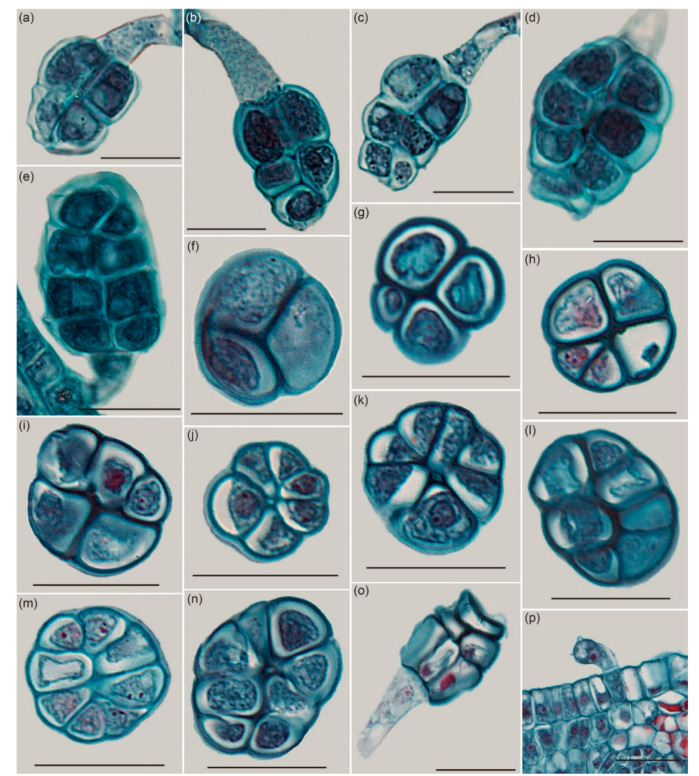
The histological structure of GTs. (**a**–**e**), (**o**) Longitudinal sections. (**f**–**n**) Transection. (**a**–**n**) Mature GTs. (**o**) A shrunken GT after secreting. (**p**) The youngest GT. Scale bars: (**a**–**p**) = 20 μm.

Based on the observation of paraffin embedding in the vertical section, a single GT consisted of two parts: the stalk, which has no cellular structure, and the head, which was composed of 4–8 cells in a longitudinal section (Figure 3a–e,o). Most GTs contained only one cell on top (head) (Figure 3b,d,e,o), but a small proportion of GTs had two cells side by side (Figure 3a,c). From the view of the transections, two types of arrangements were observed: two rows (Figure 3g,i,l,n) or a petaloid (Figure 3f,h,j,k,m). There were 3–8 cells observed in the transection (Figure 3f–n). Cells in mature GTs were full in size (Figure 3a,b,d,e), but the cells shrank and were smaller in size after secreting (Figure 3c,o). GT was unicellular at the initial stage with no apparent boundary between the stalk and the head, the base of a GT was a normal epithelial cell (Figure 3p). During the later stages, multiple layers of cells occurred and were arranged in a pyramid style.

**Figure A2:**
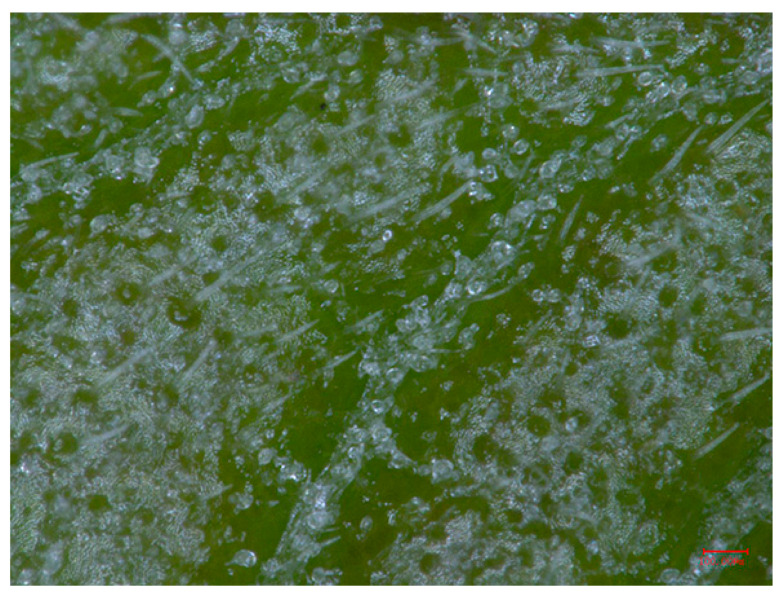
Three-dimensional photo of GTs. The GTs were half transparent.

The GTs were half-transparent in fresh leaves under the three-dimensional microscope (Figure A2).

**Figure A3:**
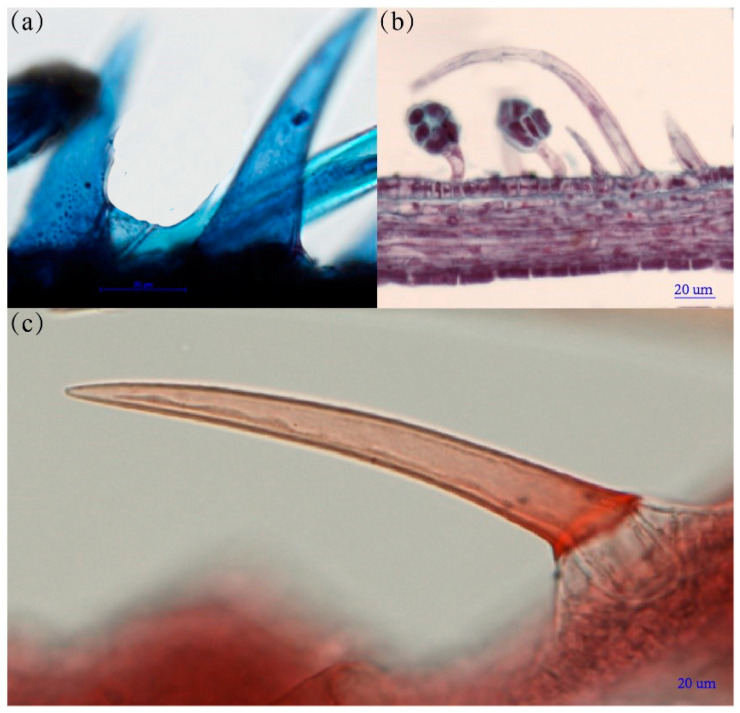
The tomenta were stained with different dyes. (**a**) The tomenta were stained as blue with Nile blue A. (**b**) The tomenta treated with paraffin. (**c**) A tomentum stained by Oil Red O. We did not observe cellular structures in the tomenta.

The tomenta were observed at the same time, but we were unable to find cells in the tomenta; there were only nonliving epidermal structures (Figure A3). 

### 2.3. Component Analysis of GTs

**Figure 4:**
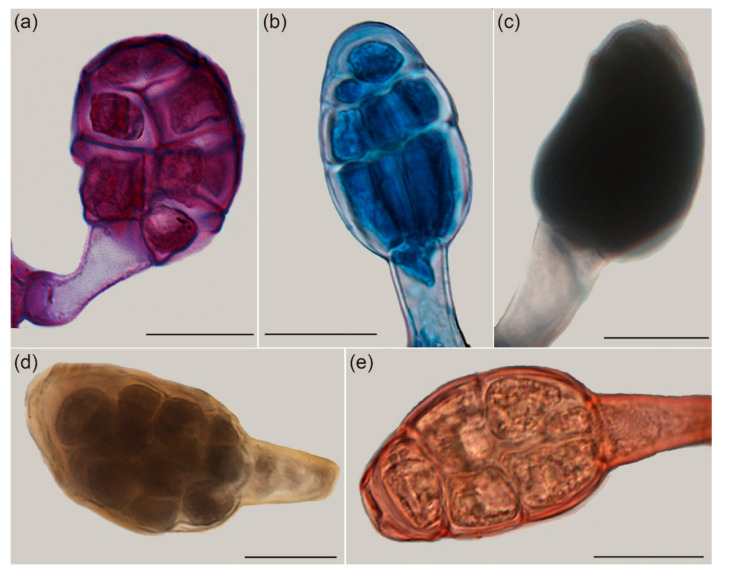
Component analysis of GTs. (**a**) A GT stained as modena with PAS (for total polysaccharides). (**b**) GT cells stained mazarine with Nile blue A (for acidic lipids). (**c**) A GT stained black with tannic acid/ferric chloride (for mucilage). (**d**) A GT stained black with ferric chloride (for phenolic compounds). (**e**) A GT weakly stained with Oil Red O (for neutral lipids). The results indicated the secretion of GTs contain a significant amount of polysaccharides, phenolic compounds, mucilage, and acidic lipids but very few neutral lipids. Scale bars: (**a**–**e**) = 20 μm.

The cells of the GTs were deeply stained with periodic acid–Schiff (PAS), Nile blue A, or ferric chloride, which produced colors of modena (Figure 4a), mazarine (Figure 4b), or black (Figure 4d), respectively. The GTs turned completely black after staining by tannic acid/ferric chloride (Figure 4c). The GTs were weakly stained with Oil Red O (Figure 4e), indicating that GTs on *R. potaninii* Maxim contain a high abundance of total polysaccharides, acidic lipid, phenolic compounds, and mucilage but a low abundance of neutral lipids.

### 2.4. Gene Expression Pattern of the GTs

The total ribonucleic acid (RNA) of different samples was extracted for RNA sequencing (RNA-seq); the results were listed in Table A1. The total RNA of GTs and leaves fulfilled the demands of RNA_seq, but a low RNA concentration and bad quality of tomenta were obtained (Table A1). Thus, we performed the RNA-seq on GTs and leaves.

**Figure A4:**
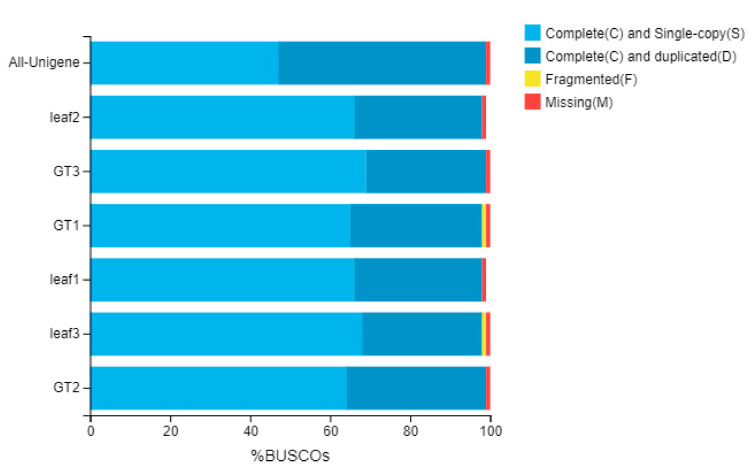
Assembly evaluation of BUSCO. The majority of unigenes were complete and single-copy or complete and duplicated; there were only a few fragmented and missing bases, i.e., the quality of the unigenes is adequate.

**Figure A5:**
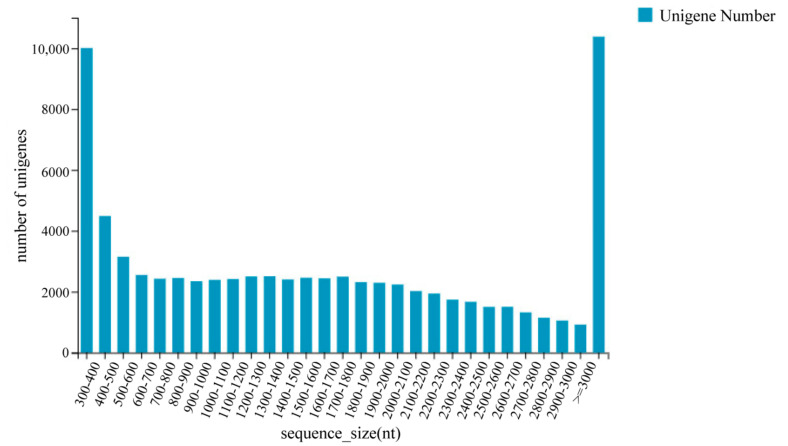
The distribution of the length of the unigenes. The length of 10,009 unigenes is 200–300 bp; there are 10,382 unigenes longer than 3000 bp, and the other unigenes are 300–3000 bp.

**Figure A6:**
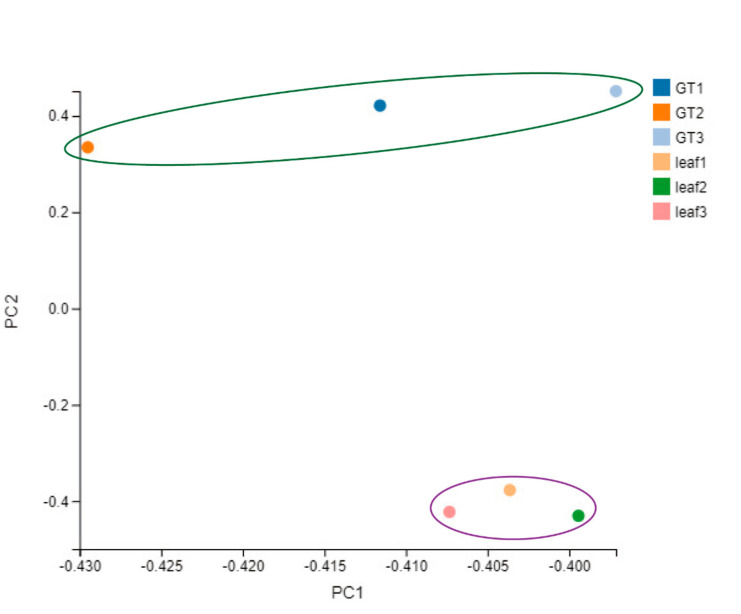
Principal component analysis (PCA) of six samples. Three leaf samples clustered and three GT samples grouped into another type.

**Figure A7:**
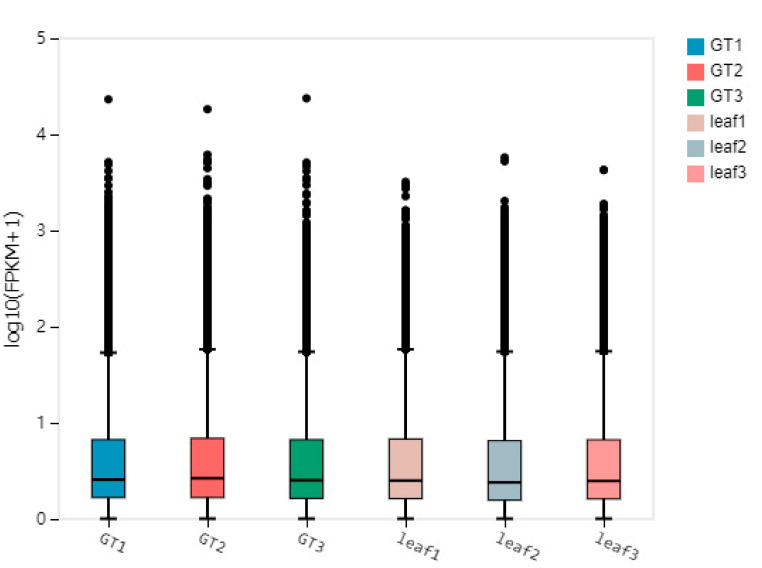
Boxplot of six samples. There are a few abnormal values in the six samples, and they show a right-skewed distribution.

**Figure A8:**
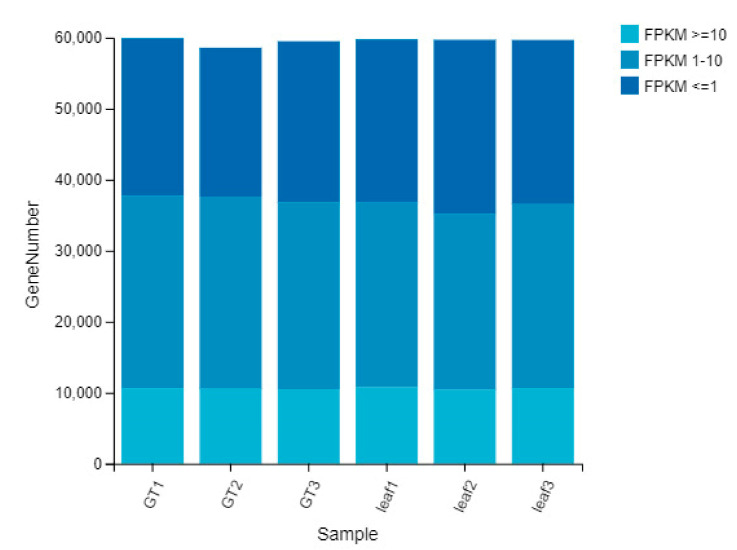
The distribution of unigenes based on FPKM. A total of 23,535 unigenes show FPKM ≤ 10; a total of 25,581 unigenes show 1 ≤ FPKM ≤ 10; and 10,616 unigenes show FPKM ≤ 1 in the leaves. A total of 21,879 unigenes show FPKM ≤ 10; a total of 26,849 unigenes show 1 ≤ FPKM ≤ 10; and 10,551 unigenes show FPKM ≤ 1 in the GTs.

Raw data were first assessed and trimmed; the results are shown in Table A2. Clean reads were assembled and then evaluated by BUSCO (Figure A4). A total of 52,601 unigenes covering a total of 127,567,799 nucleotides were identified. The length of 10,009 unigenes was 200–300 bp; 10,382 unigenes were longer than 3000 bp and other unigenes were 300–3000 bp (Figure A5). The average length of the high-quality reads was 1405 bp. The statistics of the sequence reads and assembled unigenes are listed in Table A3 and Table A4. The principal component analysis (PCA) showed three leaf samples clustered and three GT samples grouped with another type (Figure A6); the boxplot suggested that there are few abnormal values in the six samples, and unigenes are in a right-skewed distribution (Figure A7). The fragments per kilobase of exon model per million mapped fragments (FPKM) of most of the unigenes were higher than one, and the GTs and leaves showed a similar tendency (Figure A8). The unigenes were annotated into nonredundant protein sequences (Nr), nucleotide sequences (Nt), the manually annotated and curated protein sequence database (Swiss-Prot), Kyoto Encyclopedia of Genes and Genomes (KEGG), Clusters of Orthologous Groups of Proteins (COG), Pfam, and Gene Ontology (GO), and the annotated results are shown in Table A5. 

**Figure 5:**
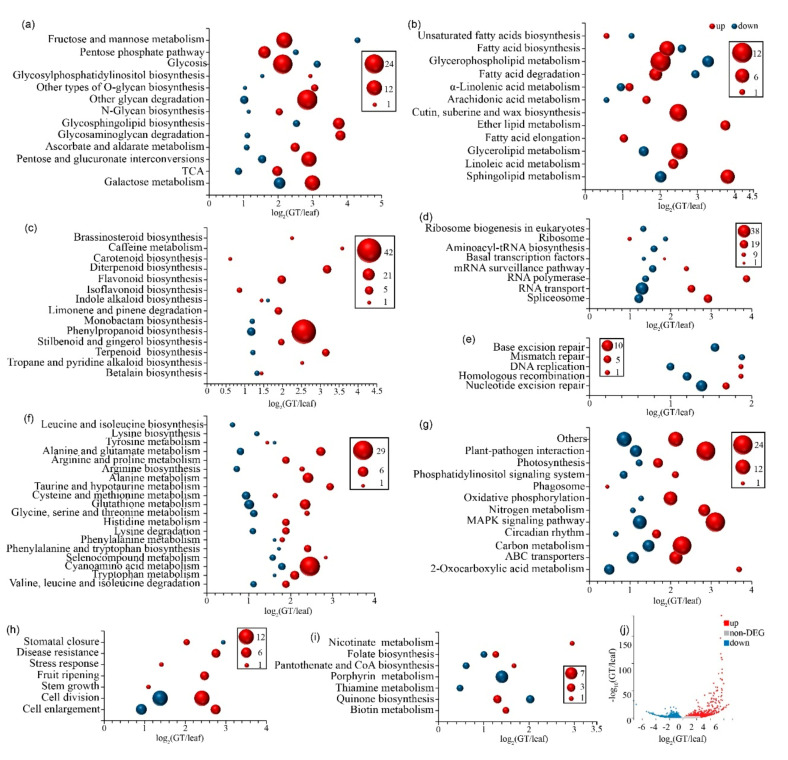
The DEGs of GTs versus leaves. (**a**) The DEGs in the pathway of carbohydrate. The majority of DEGs were enriched in glycan degradation, followed by glycolysis, fructose, and mannose metabolism. (**b**) The DEGs in the lipid pathway. There were the most DEGs in glycerophospholipid metabolism, which regulates neutral lipids; other DEGs regulate acidic lipids. (**c**) The DEGs in the pathway of secondary metabolism. The phenylpropanoid biosynthesis shows maximum upregulated DEGs and plays an important role in plant defense. (**d**) The DEGs in the subpathways of RNA biosynthesis and metabolism. All subpathways in RNA biosynthesis and transport were inhibited, and the maximum downregulated DEGs were found in RNA transport. (**e**) The DEGs in the DNA repair pathway. All subpathways were also inhibited. (**f**) The DEGs in the amino acid pathway. Cyanoamino acid metabolism was significantly upregulated, which would strengthen the plant defense systems. (**g**) The DEGs in the other pathways. All pathways were enhanced, except the phosphatidylinositol signaling system. (**h**) The DEGs in the pathway of plant hormone signal transduction. The maximum DEGs were found in cell division, followed by cell enlargement, disease resistance, and stomatal closure. (**i**) The DEGs in the pathway of cofactors. Thiamine metabolism, porphyrin, and chlorophyll metabolism were strongly inhibited, and biotin metabolism was tightly enhanced. (**j**) The volcano plot of all DEGs. In all DEGs or in a single pathway, the upregulated genes were far greater in number than downregulated genes, and the range of the upregulation was higher than that of downregulation in general, except for the cofactor. In the RNA biosynthesis and metabolism and DNA repair pathway, even though the number of downregulated genes was higher than that of upregulated genes, the range of the upregulation was also higher than that of downregulation. The *x*-axis of (**a**–**j**) is the name of the subpathways; the *y*-axis is log_2_-(GT/leaf); red means upregulated and dark blue means downregulated. The size of the bubbles indicates the number of DEGs.

**Figure A9:**
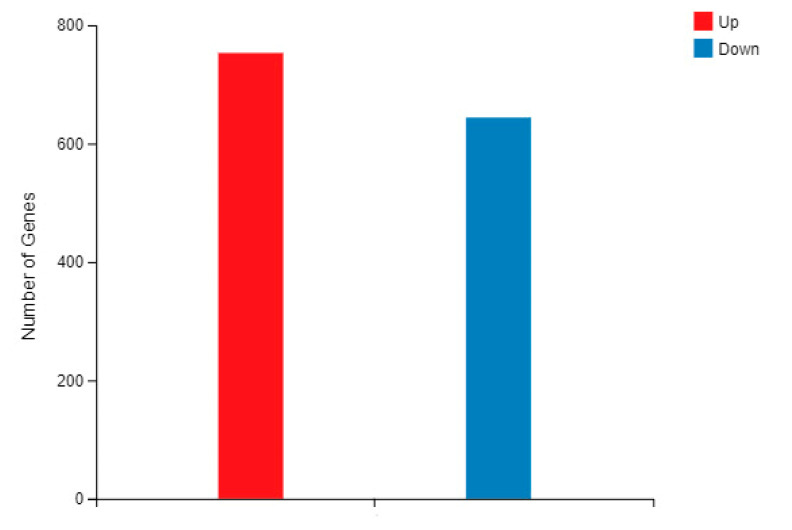
The up- and downregulated genes in GTs versus the leaves. There are 753 upregulated genes and 644 downregulated genes.

Among all unigenes, only 753 upregulated genes and 644 downregulated genes were found (Figure A9). As a whole, these differentially expressed genes (DEGs) could be divided into nine categories: carbohydrate pathway (Figure 5a), lipid pathway (Figure 5b), secondary metabolism pathway (Figure 5c), subpathways of RNA biosynthesis and metabolism (Figure 5d), deoxyribonucleic acid (DNA) repair pathway (Figure 5e), amino acid pathway (Figure 5f), plant hormone signal pathway (Figure 5g), cofactor pathway (Figure 5i), and others (Figure 5h). They had all been strengthened to varying degrees, except the RNA biosynthesis and metabolism, DNA repair pathways, and cofactors pathway (Figure 5). The majority of DEGs were enriched in the carbohydrate pathway, and upregulated genes were significantly greater in number than downregulated genes in all subpathways. The top five enriched subpathways of carbohydrate were glycan degradation, followed by glycolysis, galactose metabolism, fructose and mannose metabolism, and pentose and glucuronate interconversions (Figure 5a). In the lipid pathway, all subpathways were also enhanced, and the most enriched subpathway was glycerophospholipid metabolism, which regulates neutral lipids. The rest of the subpathways control the acidic lipid (Figure 5b). The secondary metabolism shows a similar pattern; almost all subpathways were upregulated, especially phenylpropanoid biosynthesis: a total of 42 genes were upregulated, and five genes were downregulated; it was also the top subpathway that enriched the highest number of upregulated genes. Other subpathways include diterpenoid biosynthesis, terpenoid biosynthesis, stilbenoid, diarylheptanoid, and gingerol biosynthesis, which were enhanced (Figure 5c), and all of them serve for plant defense. In the plant’s defense system, we also found other subpathways, such as plant–pathogen interaction and an enhanced mitogen-activated protein kinase (MAPK) signaling pathway in GTs (Figure 5g). In the amino acid pathway, cyanoamino acid metabolism was enhanced (Figure 5f). The plant hormone signal pathways that focus on cell division and cell enlargement were also enhanced; this may be because the GTs were developing during the sample collection (Figure 5h). 

In the RNA biosynthesis and metabolism and DNA repair and cofactor pathways, the downregulated genes were greater in number than the upregulated genes in all the subpathways (Figure 5d,e). Porphyrin and chlorophyll metabolism were also inhibited in the GTs (Figure 5i). 

Interestingly, the average range of upregulated genes is higher than downregulated genes in general, except for the cofactor pathway, regardless of how the numbers of up- or downregulated genes change (Figure 5).

### 2.5. The Selectively Expressed Genes

**Figure 6:**
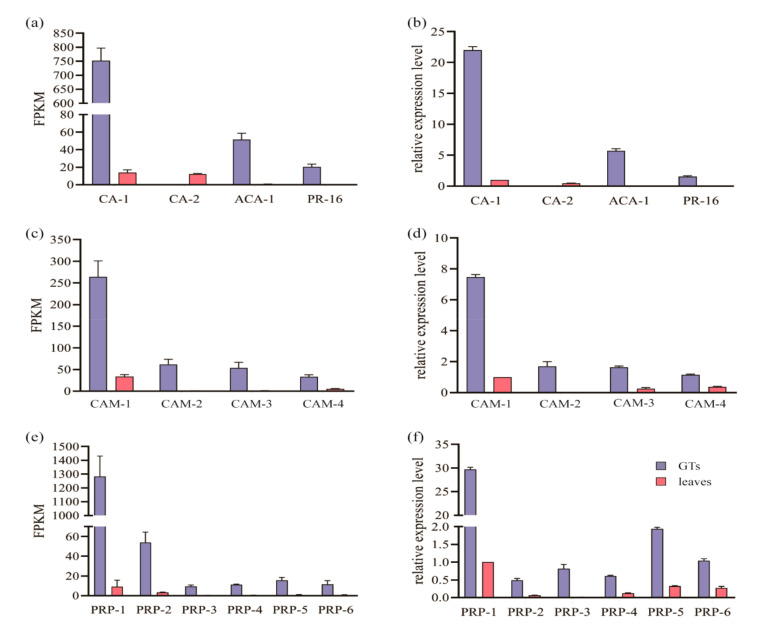
The FPKM and relative expression level of selectively expressed genes. (**a**) The FPKM of *CA-1*, *CA-2*, *ACA-1* and *PR-16* in GTs and leaves. (**b**) The relative expression level of *CA-1*, *CA-2*, *ACA-1* and *PR-16* in GTs and leaves. (**c**) The FPKM of *CAM-1*, *CAM-2*, *CAM-3* and *CAM-4* in GTs and leaves. (**d**) The relative expression level of *CAM-1*, *CAM-2*, *CAM-3* and *CAM-4* in GTs and leaves. (**e**) The FPKM of *PRP-1*, *PRP-2*, *PRP-3*, *PRP-4*, *PRP-5* and *PRP-6* in GTs and leaves. (**f**) The relative expression level of *PRP-1*, *PRP-2*, *PRP-3*, *PRP-4*, *PRP-5* and *PRP-6* in GTs and leaves. The FPKM of selectively expressed genes are shown in the left panel; the relative expression level is shown in the right panel. *ACA-1*, *PR-16*, *CAM-2*, *CAM-3*, and *PRP-3* were expressed only in GTs. *CA-1*, *CAM-1*, *CAM-4*, *PRP-1*, *PRP-2*, *PRP-4*, *PRP-5*, and *PRP-6* were detected in GTs and leaves, but the expressed level in GTs was significantly higher than in leaves. *CA-2* could be detected only in leaves. The transcriptome and RT-qPCR showed a similar tendency.

We selected 13 unigenes selectively expressed in GTs which are responsible for regulating chitinase activity (*CA-1*), carbonic anhydrase (*ACA-1*), PR domain zinc finger protein 16 (*PR-16*), cyanoamino acid metabolism (*CAM-1*, *CAM-2*, *CAM-3*, *CAM-4*), and pathogenesis-related proteins (*PRP-1*, *PRP-2*, *PRP-3*, *PRP-4*, *PRP-5*, *PRP-6*). *ACA-1*, *PR-16*, *CAM-2*, *CAM-3*, and *PRP-3* were expressed in the GTs only; other genes were detected in both GTs and leaves, but the FPKM of GTs were significantly higher than that of leaves. *CA-2* showed a different pattern: it was detected only in the leaves. Quantitative real-time polymerase chain reaction (RT-qPCR) was performed to verify the reliability of the results. The primers used in this study are listed in Table A6. The RT-qPCR showed the same tendency among all unigenes; only *ACA-1*, *PR-16*, *CAM-2*, and *PRP-3* were detected in GTs; *CA-1*, *CAM-1*, *PRP-1*, *PRP-5,* and *PRP-6* were detected both in GTs and leaves, but the expression level in GTs was significantly higher than in leaves (Figure 6).

## 3. Discussion

Different types of GTs have been reported in different plant species, including bulbous trichomes (lamp bulb-like) [5], capitate-sessile trichomes, capitate-stalked trichomes, and peltate trichomes [16]. On the leaves of *R. potaninii*, only one type of GT was observed. The GTs on *R. potaninii* leaves look like grenades, with a slender (noncellular) stalk and an expanded head with 8–15 cells. It was observed that during development, GTs have initially only one cell, and the cell divides into multiple cells in mature GTs. Cells in the mature GTs were full of cell content, but these cells became thinner and shrank after secreting. 

The distribution and density of GTs and nonglandular trichomes were distinctly different. In young leaves, the density of GTs was greater and decreased during leaf development. During the fourth and fifth leaf stages, GTs and tomenta in the chamber of the adaxial side disappeared, indicating that they had disappeared completely in the third stage. However, some GTs and tomenta were still found in the regions close to the vein. In general, the density of tomenta was far higher than that of GTs in the same regions during the same stages of leaf development. In the abaxial side of leaves, the density of tomenta was far higher than that of GTs during the first stage of the leaves. The density of tomenta decreased substantially starting from the third stage, with no tomentum visible during the fifth stage. GTs decreased during leaf development but at a slower pace. During the fifth stage, there were still a certain number of GTs visible on the abaxial side of the leaf. Based on our observation, we assume that GTs and tomenta are involved in defense mechanisms against sucking insects. During the early development stages of the leaf, the high density of GTs and tomenta can offer more effective chemicals and physical barriers for defense. During the development of the leaves, a firmer cuticle and thicker wax coat are formed on the leaf surface, which protect leaves from drought and pests and results in a reduced requirement of GTs and tomenta [17].

The secretion from GTs on the leaves of *R. potaninii* is very little, making it difficult to collect and analyze its composition by analytical quantification methods. Hence, as far as we know, no records have been reported about the components of the secretion of GTs on *R. potaninii* leaves. Accordingly, these secretions were evaluated via staining with different dyes. Based on our staining estimation, the secretion from GTs contains polysaccharides, phenolic compounds, mucilage, and acidic lipids; interestingly, it also consists of a small concentration of neutral lipids. The secretion formed from these chemicals will protect developing leaves from drought due to its notable water absorption capacity and water-retaining ability [18,19].

The highlight of this paper concerns the gene expression pattern of GTs; the results indicated that a gene shift happened, and it was consistent with the staining. The glycerophospholipid metabolism was enhanced; thus, few neutral lipids were found in the GTs, likely because the acidic lipid would provide better protection for young leaves, such as inhibiting some basophilic bacteria or fungi. Mucilage can provide sufficient adhesion, and lipids in the secretion act as a lubricant, which is beneficial in reducing potential friction among young leaves during their early expansion and development [20]. At the same time, the phenylpropanoid and phenolic biosynthesis, plant–pathogen interaction pathway, and cyanoamino acid metabolism were all enhanced significantly. A reasonable explanation is that phenylpropanoids in the stem are involved in the shikimate pathway, which is the precursor to simple phenols, flavonols, isoflavonols, etc. Phenolic compounds can be produced from chemical barriers, which can fend off microorganisms and other pests [21]. Furthermore, four unigenes which regulate the cyanoamino acid metabolism were only expressed in the GTs. Cyanoamino acid is an amino acid derivative that contains a cyanide group and is toxic to insects; thus, it acts as a protective substance in plants which has a toxic effect on the feeder [22]. The greatest change happened in carbohydrate; glycan degradation was enhanced significantly. The metabolism of monosaccharides, disaccharides, pentose, and glucuronate interconversions were also vigorous. This supports the strong cellular activity and materials conversion. Relatively high amounts of polysaccharides have been found in many types of secretions [23], including secretions from colleters on *Anacardium humile*, *Lithraea molleoides*, *Spondias dulcis,* and *Tapirira guianensis* trees. Colleters are a different type of plant structure but could play defense roles similar to GTs [24]. The exact function of polysaccharides in the secretions of GTs remains to be delineated.

It is worth noting that whether the number of DEGs was large or small, the mean value of log_2_(GT/leaf) of upregulated genes was greater than the mean value of log_2_(GT/leaf) of downregulated genes in most subpathways, which indicates that upregulation was higher than downregulation in GTs. This differs from the DEGs in the peltate GTs of *Mentha spicata*; the log_2_-fold changes of downregulated genes were significantly higher than upregulated genes [25]. We suggest the differences in gene expression between different species are due to their performing specific functions. 

Furthermore, we found some selectively expressed genes in GTs. Most of these genes regulate the plant defense pathways, such as *CA-1* regulating chitinase activity. Plant chitinase antagonizes the hyphae growth of more than 20 pathogenic and nonpathogenic fungi, such as chitinase inhibiting the spore germination of *Rhizoctonia solanacearum*. In addition to antifungal properties, chitinase also showed some resistance to bacteria, insects, and mites [26]. Six selectively expressed genes regulate the pathogenesis-related protein in response to infection by pathogens such as fungi or viruses [27]. Pathogenesis-related protein is also involved in salicylic acid-mediated disease resistance [28]. 

GTs are the first contact point with surroundings; they are essential for the maintenance of physiologically favorable conditions [29]. Except in response to biotic stress, GTs also play an important role in response to abiotic stress. GTs can affect leaf temperature, photosynthesis, and water loss by increasing light reflectance [30]. In the leaves of tomato, the ratio of trichomes to stomata is associated with water use efficiency [29]. 

When we collected the GTs for RNA-seq, the samples inevitably mixed with tomenta. This did not affect the results of the RNA_seq of the GTs because there were no cells in the tomenta; thus, we were unable to extract the total RNA from the tomenta (Figure A3).

In summary, we examined the distribution of GTs and tomenta on both the adaxial and abaxial sides of the leaves of *R. potaninii* during leaf development. In addition, we analyzed the gene expression pattern of the GTs. We found that the overall patterns of changes in both the GTs and tomenta were similar, but the density of GTs was reduced at a slower pace during leaf development. Moreover, the trichomes comprise noncellular stalks and an expanded head with 8–15 cells. The cells in trichomes became smaller and shrank after secretion. Secondary metabolites, such as phenolics in the secretion of trichome secretions, are consistent with their defensive roles for young leaves. The gene expression showed a similar pattern to that of the staining: the carbohydrate, lipid, amino acid (especially toxic amino acid), and phenylpropanoid pathways were all enhanced, and the DEGs of GTs were shifted and selectively expressed to serve for specific functions.

## 4. Materials and Methods

### 4.1. Materials

**Figure 7:**
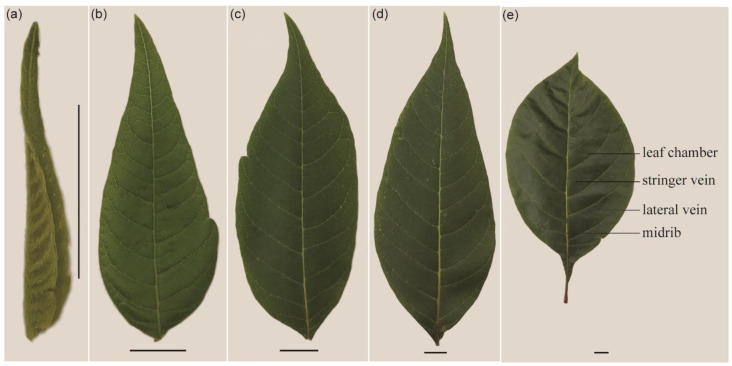
Leaves of *R. potaninii* Maxim during different stages. (**a**) A leaf during the first stage of development. (**b**) A leaf during the second stage of development. (**c**) A leaf during the third stage of development. (**d**) A leaf during the fourth stage of development. (**e**) A leaf during the fifth stage of development, and the positions of structures that were used in this paper. Scale bars of all pictures are 1 cm.

Six-year-old *R. potaninii* plants were grown in an experimental field at the Research Institute of Resources Insects of Kunming, Yunnan province, southwest China. Leaves of *R. potaninii* plants were classified as initial stage when they were 1.8 mm; then, the leaves were collected once a week and defined as second to the fifth stage in turn (Figure 7). The leaves for the different tests came from three randomly selected trees.

### 4.2. Paraffin Sections

The fresh leaves of each stage were fixed in a formaldehyde-acetic acid-ethanol fixative (FAA) solution containing 5 mL of formaldehyde, 5 mL of acetic acid, and 90 mL of 70% ethyl alcohol for two days. The samples were then cut into 2–3 mm pieces and dehydrated in a series of ethanol solutions (70% ethanol for 1 h, 80% ethanol for 45 min, 90% ethanol for 30 min, 95% ethanol for 15 min, 100% ethanol for 10 min). The processed samples were embedded in paraffin; 8 μm-thick sections were made using a rotary microtome (Leica RM2126RT, Solms, Hesse-Darmstadt, Germany). The sections were deparaffinized and stained with safranin and fast green for measuring the histological structure or stained with PAS for total polysaccharides [31]. The sections were tested under a light microscope (Nikon LV150N, Tokyo, Japan). Five leaves were used during the initial stage, and another five samples were used during the fourth stage.

### 4.3. Frozen Sections

The fresh leaves were embedded in Tissue OCT-Freeze medium (APPLYGEN, Beijing, China) and cut into 20 μm sections with a freezing microtome (Leica CM1950, Solms, Hesse-Darmstadt, Germany); these sections were further stained with tannic acid/ferric chloride (Sangon Biotech, Shanghai, China) for mucilage [32], using Nile blue A (Sangon Biotech, Shanghai, China) for acidic lipids [33], ferric chloride (Sangon Biotech, Shanghai, China) for phenolic compounds [34], and Oil Red O (Sangon Biotech, Shanghai, China) for neutral lipids [35]. Standard control procedures were carried out simultaneously. Each staining method was repeated with three leaves; each leaf consisted of more than 20 GTs. The sections were tested under a light microscope (Nikon LV150N, Tokyo, Japan).

### 4.4. Scanning Electron Microscope (SEM)

The fresh samples were fixed in 4% glutaraldehyde for 2 h, then dried in the air and sprayed gold. The density and appearance of GTs were observed using an SEM (Tabletop Microscope 3000, Tokyo, Japan). MANOVA was performed using SPSS 20.0 for analysis of the number of GTs and tomenta; the stage was a fixed variate, and the different leaf structures were a dependent variable, *p* < 0.01. We counted 10 leaves, which were collected from three trees randomly at every stage; every leaf was detected one time for the same type of region.

### 4.5. Three-Dimensional Microscope

The fresh leaves (initial stage) were used for observing the three-dimensional image of GTs directly by the three-dimensional microscope (MSD-VHX1000, Tokyo, Japan). 

### 4.6. Transcriptome Sequencing

GTs and tomenta were shaved gently from fresh leaves during the initial stage of leaves by a sharp blade under an anatomic microscope (Nikon SMZ800, Tokyo, Japan). The collected GTs, tomenta, and shaved leaves were placed into the RNA stores (Takara, Dalian, China) immediately, respectively. Then, the samples were stored at −80 °C for later use. Tomenta and mature GTs in every sample were collected from 20 leaves during the initial stage; the 20 shaved leaves were mixed as single leaf sample. There were three replicates for every treatment.

Tissues of 1 mg were used for RNA extraction (BioTeke Corporation, Beijing, China). The total RNA was purified and assessed by an RNA Nano 6000 Assay kit (Agilent Technologies, Palo Alto, CA, USA). First-strand cDNA was synthesized with random hexamers as primers; then, double-stranded cDNA was synthesized and purified by using a QiaQuick PCR Extraction kit (QIAGEN, Dusseldorf, North Rhine-Westphalia, Germany). Sequencing adaptors were ligated to fragments and further purified by agarose gel electrophoresis and enriched by PCR amplification. Sequencing libraries were generated using a Next Ultra Directional RNA Library Prep Kit from Illumina (New England Biolabs, MA, USA). After cluster generation, the libraries were sequencing using the Illumina HiSeq 2000 platform (BGI, Shenzhen, China), and paired-end reads were generated. Finally, products were purified with the AMPure XP system (Beckman Coulter, Brea, CA, USA), and library quality was confirmed on the Agilent 2100 Bioanalyzer (Agilent Technologies, Palo Alto, CA, USA).

Raw data were assessed by SOAPnuke 1.4.0, (BGI, Shenzhen, China); then, adaptors and reads with more than 5% unknown bases and more than 20% of the bases with a quantity value less than 15 were assessed by trimmomatic 0.36 with ILLUMINACLIP:2:30:10 LEADING:3 TRAILING:3 SLIDINGWINDOW:4:15 MINLEN:50. Transcriptome assembly was accomplished using Trinity with default parameters [36,37]. 

### 4.7. Gene Annotation

Gene function of all unigenes was annotated into the GO and KEGG database using the online platform David: https://david.ncifcrf.gov/summary.jsp (accessed on 7 July 2020), Nt, Nr, Swiss-Prot, and COG database with blast+ 2.9.0. The parameters were the default ones. The thresholds for GO terms and homolog identification were *p*-adjusted < 0.05. Then, the annotations were merged into a csv file using V-look. 

### 4.8. DEGs

The FPKM of all unigenes in all sample were calculated by DESeq2 with estimation of size factors (estimateSizeFactors), estimation of dispersion (estimateDispersons), negative binomial GLM fitting, and Wald statistics (nbinomWaldTest). The negative binomial distribution principle of DESeq2 was used for the identification of DEGs at *p*-value ≤ 0.05. The gene was marked as downregulated if log_2_ (GT/leaf) < 0 and marked as upregulated if log_2_(GT/leaf) > 0 at *p* ≤ 0.01 [38,39]. The adjusted *p*-value was < 0.05. The expressions of DEGs were analyzed by SPSS 20.0 (IBM, Armonk, NY, USA). To analyze the specific pathways of GTs, the average log_2_ (GT/leaf) of DEGs in key subpathways was calculated. If two thirds of DEGs were upregulated in a pathway, they were marked as upregulation, and vice versa. 

### 4.9. Validation of Transcriptomic Data via RT-qPCR

To verify the significance of transcriptomic analysis results, the DEGs were selected and validation was carried out via RT-qPCR. The primers were designed by Primer-primer 6.0 (Table A6); we chose *tubulin* as a reference gene and leaves as the control. The GTs and leaves were collected as mentioned above, and total RNA was extracted by the TaKaRa MiniBEST Plant RNA Extraction Kit (TaKaRa, Dalian, China) following the manufacturer’s instructions. The first-strand cDNA was produced by the PrimeScript™ RT reagent Kit with gDNA Eraser (Perfect Real Time) (TaKaRa, Dalian, China). The PCR was amplified in a 25 μL reaction mixture (1 μL sense primer (0.5 μM), 1 μL antisense primer (0.5 μM), 1 μL template (100 ng\μL), and 22 μL master mix) in QuantStudio 7 Flex (Thermo Fisher, Waltham, MA, USA): initial denaturing step at 95 °C for 4 min, followed by 40 cycles of denaturing at 94 °C for 1 min, annealing at 53 °C for 1 min, and extension at 72 °C for 30 sec. Then, the relative expression levels of DEGs were calculated based on the CT values [40].

## Data Availability

The data that support the findings of this study are openly available in the NCBI Sequence Read Archive (SRA) under project PRJNA662477 [https://www.ncbi.nlm.nih.gov/bioproject/PRJNA662477]. Accessed on 9 September 2020.

## Appendix A

**Table A1:** Quality evaluation of the glandular trichome, tomentum, and leaf.

Sample	Concentration (ng/μL)	Volume (μL)	Total RNA (μg)	OD260/280	OD260/230	RIN	28S/18S
GT-1	151	30	4.53	1.98	1.18	8.9	1.5
GT-2	79	15	1.19	2	0.61	9.0	2.9
GT-3	143	15	2.15	2.04	0.71	9.3	2.6
Tomentum-1	18	15	0.27	1.86	0.14	8.5	2.8
Tomentum-2	26	15	0.39	1.91	0.69	8.8	2.8
Tomentum-3	0.225	15	0.0034	2.11	0.08	2.4	0.0
Leaf-1	2556	30	76.68	2.03	2.08	9.7	1.9
Leaf-2	2168	30	65.04	1.98	1.93	9.0	1.6
Leaf-3	3070	30	92.1	1.91	2	9.6	1.8

**Table A2:** Quality evaluation of clean reads.

Sample Name	Total_Raw _Read (M)	Total_Clean _Read (M)	Total_Clean _Base (Gb)	Clean_Read _q20 (%)	Clean_Read _q30 (%)	Clean_Read _Ratio (%)
GT-1	43.82	43.39	6.51	97.56	93.32	99.01
GT-2	43.82	43.43	6.51	97.57	93.3	99.11
GT-3	43.82	43.41	6.51	97.62	93.44	99.06
Leaf-1	43.82	43.42	6.51	97.51	93.18	99.08
Leaf-2	43.82	43.27	6.49	97.67	93.59	98.73
Leaf-3	43.82	43.35	6.5	97.55	93.28	98.93

Total Raw Reads(Mb): number of reads before filtering; Total Clean Reads(Mb): number of reads after filtering; Total Clean Bases(Gb): the total base amount after filtering; Clean Reads Q20(%): the rate of bases whose quality is greater than a value of 20 in clean reads; Clean Reads Q30(%): the rate of bases whose quality is greater than a value of 30 in clean reads; Clean Reads Ratio(%): the ratio of the number of clean reads.

**Table A3:** Statistics of transcriptome.

	Total Number	Total Length (bp)	Max Length (bp)	Min Length (bp)	Mean Length (bp)	N50	GC%
Unigene	52,601	62,505,864	15,303	297	1405	1497	42.71

Total Number: the total number of transcripts; Total Length: the read length of transcripts; Max Length: the max length of transcripts; Min Length: the min length of transcripts; Mean Length: the average length of transcripts; N50: the N50 length is used to determine the assembly continuity; the higher, the better; N50 is a weighted median statistic: 50% of the total length is contained in unigenes that are equal to or larger than this value; GC(%): the percentage of G and C bases in all transcripts.

**Table A4:** Quality metrics of Unigenes.

Samples	Total Number	Total Length (bp)	Mean Length (bp)	N50 (bp)	N70 (bp)	N90 (bp)	GC (%)
GT-1	51,661	71,811,850	1390	2033	1466	732	40.02
GT-2	50,439	65,672,100	1302	1909	1383	674	40.14
GT-3	48,387	65,638,323	1356	1998	1432	706	40.19
Leaf -1	47,472	67,237,102	1416	2055	1490	749	40.17
Leaf -2	49,157	65,750,946	1337	2016	1439	684	40.19
Leaf -3	49,113	68,843,747	1401	2060	1479	733	40.07

Sample: sample name; Total Number: the total number of unigenes; Total Length: the read length of unigenes; Mean Length: the average length of unigenes; N50: the N50 length is used to determine the assembly continuity; the higher, the better; N50 is a weighted median statistic: 50% of the total length is contained in transcripts that are equal to or larger than this value. N70: similar to N50; N90: similar to N50; GC(%): the percentage of G and C bases in all unigenes.

**Table A5:** Annotation result statistics in different databases.

Database	Numbers of Unigene	Percent (%)
Total	79,032	100
Nr	61,027	77.22%
Nt	52.369	66.26%
Swiss-Prot	46,693	59.08%
KEGG	49,677	62.86%
KOG	48,497	61.36%
Pfam	48,645	61.55
GO	46,304	58.59%
Intersection	26,043	32.95%
Overall	62,877	79.56

Intersection: The number of unigenes annotated by all seven functional databases; Overall: the number of unigenes annotated by any of the seven functional databases.

**Table A6:** Primers in this study.

Genes	Sense Primer	Anti-Sense Primer
*tubulin*	TGCTCTTCTGAAACACCCTTGA	CCAACTGGGCTGAGAACTAACA
*CA-1*	CGGTGATGACAGCGTGACA	CCAGCAGGAAGAGCCATTAAC
*CA-2*	AAGCCAAGTGCCTCTCATCA	TTACCTTCCTTGTATTCCTTCATCC
*ACA-1*	TGCTTCTGGTTGTTGCTTCC	GTTGACTGGAGATTGGTGCTT
*PR-16*	GATAAGAATGCCGACGACAGATT	CACAACACGCTCAAGATTGGA
*ACM-1*	TGGTAGAGAATACTGGCGAGAT	CTCAACGGACTCAGCAACTAG
*ACM-2*	ACGCCGAATGTGTCAGGTT	GGATAAGCATCAGATGGTTCAGAA
*ACM-3*	TGCCTGTATCAACTATGGAGTCT	CGCTCTTCTTCTTCCTCTTCTTC
*ACM-4*	TTCGCTTCAGCAGGAATAGGA	CCACCAACAGTTATGAGTACAAGA
*PRP-1*	CGCAAGTCGGTTCGCATT	GCAATCATCTCTTATTCGGTGAC
*PRP-2*	CTCCCAAAGTCAACGGCATT	CGGTTCAACGGTGAGATTCC
*PRP-3*	TTCGCTGACATCCACTGAAGA	AGATGCTGCCACTTGAGGTT
*PRP-4*	GAAGGAATGTGAAGCCGAAGA	TTGAAGGAGGTTGACGAATGAA
*PRP-5*	GCCAAGGAGCAACTATGAATGA	TTCGTAGGTCGTAATCAGAATCG
*PRP-6*	TTCAACGACCAGCACGAGTA	CCTTCATTCCTCTTCAGCATCTC
